# Raman Microspectroscopy of Individual Algal Cells: Sensing Unsaturation of Storage Lipids *in vivo*

**DOI:** 10.3390/s100908635

**Published:** 2010-09-17

**Authors:** Ota Samek, Alexandr Jonáš, Zdeněk Pilát, Pavel Zemánek, Ladislav Nedbal, Jan Tříska, Petr Kotas, Martin Trtílek

**Affiliations:** 1 Institute of Scientific Instruments of the AS CR, v.v.i., Academy of Sciences of the Czech Republic, Kralovopolska 147, 61264 Brno, Czech Republic; E-Mails: sasa@isibrno.cz (A.J.); pilat@isibrno.cz (Z.P.); pavlik@isibrno.cz (P.Z.); 2 Institute of Systems Biology and Ecology of the AS CR, v.v.i., Academy of Sciences of the Czech Republic, Zámek 136, 37333 Nové Hrady, Czech Republic; E-Mails: nedbal@greentech.cz (L.N.); triska@usbe.cas.cz (J.T.); kotas@usbe.cas.cz (P.K.); 3 Photon Systems Instruments, Drásov 470, 664 24 Drásov, Czech Republic; E-Mail: martin@psi.cz

**Keywords:** Raman spectroscopy, algal cells, lipids, iodine value

## Abstract

Algae are becoming a strategic source of fuels, food, feedstocks, and biologically active compounds. This potential has stimulated the development of innovative analytical methods focused on these microorganisms. Algal lipids are among the most promising potential products for fuels as well as for nutrition. The crucial parameter characterizing the algal lipids is the degree of unsaturation of the constituent fatty acids quantified by the iodine value. Here we demonstrate the capacity of the spatially resolved Raman microspectroscopy to determine the effective iodine value in lipid storage bodies of individual living algal cells. The Raman spectra were collected from three selected algal species immobilized in an agarose gel. Prior to immobilization, the algae were cultivated in the stationary phase inducing an overproduction of lipids. We employed the characteristic peaks in the Raman scattering spectra at 1,656 cm^−1^ (*cis* C═C stretching mode) and 1,445 cm^−1^ (CH_2_ scissoring mode) as the markers defining the ratio of unsaturated-to-saturated carbon-carbon bonds of the fatty acids in the algal lipids. These spectral features were first quantified for pure fatty acids of known iodine value. The resultant calibration curve was then used to calculate the effective iodine value of storage lipids in the living algal cells from their Raman spectra. We demonstrated that the iodine value differs significantly for the three studied algal species. Our spectroscopic estimations of the iodine value were validated using GC-MS measurements and an excellent agreement was found for the *Trachydiscus minutus* species. A good agreement was also found with the earlier published data on *Botryococcus braunii.* Thus, we propose that Raman microspectroscopy can become technique of choice in the rapidly expanding field of algal biotechnology.

## 1. Introduction

Photosynthetic organisms transform the energy of solar photons into the free energy of chemical bonds that provide for nearly the entire energy supply of Earth’s biosphere. Annually, photosynthesis accounts for net primary production of *ca.* 56.4 × 10^15^ g of carbon assimilated from the atmosphere on land and of *ca.* 48.5 × 10^15^ g in the ocean [1]. Most of the ocean photosynthesis occurs in planktonic algae. This enormous capacity of algae to transform the solar radiation into energy-rich compounds and to remove CO_2_ from the atmosphere justifies the current interest of science and industry. Algae are considered as a potent source of biofuels of higher generation that will not compete for land with food production and that will contribute to biological capture of atmospheric CO_2_ to mitigate the global climate change.

In parallel to hydrogen and alcohols, the most often considered products from algae for the fuel industry are algal lipids [2]. Typical storage lipids in algae are triacylglycerols: tri-esters of glycerol with saturated or unsaturated fatty acids. In this paper, we focus on the degree of fatty acid unsaturation which is the key parameter that determines the application potential for fuels or as dietary supplements or for pharmaceutical raw materials.

The analysis of the fatty acid composition in algae by gas chromatography-mass spectrometry (GC-MS) has revealed a significant variability among algal species [3,4]. GC-MS is a powerful analytic technique requiring the cell disintegration prior the analysis. However, for the purpose of selection and generation of potent production strains, one needs to characterize the lipids non-invasively in living algal cells so that they can be classified and sorted for further cultivation. Vital staining by BODIPY 505/515 [5] or by Nile Red [6] are currently considered for fluorescence-activated cell sorting of lipid-rich algal cells.

Raman spectroscopy offers an attractive alternative for lipid detection that has not yet been sufficiently exploited in algae. So far, Raman applications in microbiology have aimed mostly at detecting medically relevant organisms [7–9]. In 2007, the Raman Research Group at Gent University published a database of Raman spectral features of biologically relevant molecules that facilitates assignment of the most prominent Raman bands observed in living cells [10]. Recent reviews summarize the use of Raman spectroscopy for the detection and identification of important molecules in biological samples [11–13]. Raman spectroscopy of photosynthetic organisms is complicated by a strong autofluorescence of pigments that obscures the characteristic Raman spectral features. This challenge has limited the application to only a small number of algal species [14–16].

In this paper, we present Raman spectra of storage lipid bodies measured with Raman microspectroscopy in individual cells of three algal species: *Botryococcus sudeticus*, *Chlamydomonas sp*., and *Trachydiscus minutus*. The main driving force behind the selection of the three algal species was to estimate the applicability of the spectroscopic measurements for lipid characterization in species with significantly different relative content of unsaturated fatty acids. This is exemplified for *Trachydiscus minutus* which contains a high amount of highly unsaturated fatty acids [17] that can be used as a valuable supplement of human diet. The other two algal species have been for a long time in the focus of an intense research so that the comparison with published results can be readily made [18].

The intensities of the Raman spectral peaks that correspond to the saturated and unsaturated carbon-carbon bonds in lipid molecules were used to estimate the degree of unsaturation in the lipid bodies similarly to [19–21]. The degree of unsaturation was quantified using the iodine value that is widely applied in biofuel and food industry [22]. We propose that the non-invasive single-cell sensing of the iodine value based on spatially resolved Raman microspectroscopy has a substantial potential to contribute to breeding of novel algal strains for production of higher generation biofuels and dietary supplements.

## 2. Materials and Methods

### 2.1. Organisms and cultivation conditions

*Botryococcus sudeticus* Lemmermann, CCALA 780 (VAZQUEZ-DUHALT/UTEX 2629), *Chlamydomonas sp*. CCALA, and *Trachydiscus minutus* (Bourrelly) Ettl, CCALA, were obtained from the Culture Collection of Autotrophic Organisms, CCALA (Institute of Botany, Academy of Sciences of the Czech Republic). *T. minutus* was cultivated in 50% Šetlík-Simmer medium [23] in 100 mL air-bubbled batch cultures. The irradiance during the cultivation was 400 μmol(photons)·m^−2^·s^−1^ and temperature was 28 °C. The cells were harvested in early stationary phase. *Chlamydomonas sp*. and *Botryococcus sudeticus* were cultivated in 150 mL Erlenmeyer flasks in BBM medium at room temperature in daylight at a laboratory window with occasional manual mixing. The cells were harvested at late stationary phase. The long term cultivation in the stationary phase was observed to induce the deposition of storage lipids in algal cells.

### 2.2. Nile Red staining and fluorescence microscopy

**Figure 1.**
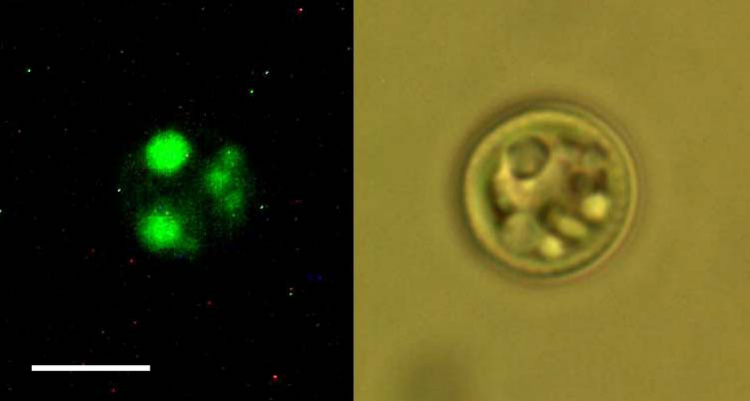
Visualization of the lipid bodies in algal cells. Nile Red (NR) fluorescence image of the lipid bodies (left) and Differential Interference Contrast (DIC) image (right) of the same living cell are compared. Complex internal compartmentalization is visible in the DIC image and the structures corresponding to the lipid bodies can be clearly identified. The scale bar is 5 μm.

The technique of vital Nile Red staining was used in our study in order to visualize lipid bodies within the algal cells. This allowed us to identify the lipid bodies in the studied cells according to their morphology and size (see Figure 1). Consequently, the lipid bodies were targeted by the focused laser beam and as a result one observes Raman scattering. Nile Red (9-diethylamino-5*H*-benzo[α]phenoxazine-5-one) was prepared according to Greenspan *et al.* [24]. The staining was performed by mixing the solution of the dye (5mg/mL in acetone) directly with the culture suspended in the medium in 1:50 ratio (24–26). DMSO (dimethyl sulfoxide) was added in 1:50 ratio to enhance the permeation [6]. Staining was applied at room temperature for a period of 30 minutes, the mixture was occasionally shaken. Cells were observed using Olympus BX50 microscope equipped with 100W high pressure mercury lamp for excitation, GFP fluorescence filter set (MDF-GFP, Thorlabs), and Olympus UPlanFl 60x / 1,25NA, oil immersion Ph3 objective lens. Pictures were acquired with Tucsen 3MPx color ½“ CMOS USB camera (Fuzhou Tucsen Imaging Technology Co., Ltd.).

### 2.3. Gas chromatography-mass spectrometry technique

*Biomass harvest and lipid extraction*––at the end of the cultivation, algal biomass, chlorophyll content, and cell counts were determined and the exact volume of algal biomass was filtered using a glass fiber filter MN GF1 (Macherey Nagel, Germany). After filtration, the filter with wet algal biomass was folded, put into a 4 ml vial and lyophilized overnight. After lyophilization the filter was wetted with acetone and the closed vial was kept at 50 °C for 30 minutes. Then the filter was spiked with hexane solution of nonadecanoic acid as internal standard for final quantification of fatty acids methyl esters, the solvent was evaporated by a gentle flow of nitrogen and the filter was cut into pieces which were placed into 25 mL zirconium oxide grinding jar and milled two times for 75 sec. After finishing the milling procedure, the resulting mixture was quantitatively transferred with chloroform into a 7 mL vial, the solvent was than evaporated to dryness and the rest was extracted three times with chloroform:methanol (2:1 v/v). Prior to the first extraction the vial was kept at 60 °C for 30 minutes with occasional shaking. After heating, the mixture was washed with potassium chloride solution (0.88% w/w), the organic layer was separated and the biomass was extracted twice using the same procedure. Chloroform extracts were evaporated to dryness by nitrogen flow. 1.5 mL of 3N hydrochloric acid in methanol was added and the reaction mixture was kept at 60 °C. After 90 minutes the reaction mixture was exhaustively extracted three times with 2.5 mL of hexane. Joint extracts were filtered through a short column filled with anhydrous sodium sulphate and evaporated again to dryness by nitrogen. The resulting product was dissolved in 1 mL of hexane and 1 μL of the hexane extract was injected into a GC-MS instrument. The extraction and trans-esterification efficiency was tested on cholesteryl linoleate (Sigma-Aldrich, Czech Republic).

*GC-MS analyses* - an ITQ 1100 instrument (Thermo Fisher Scientific, U.S.A.) was used, equipped with a PTinjector operated in splitless mode with the injection temperature set to 250 °C. The GC separation was performed with a Zebron ZB-5 column of 30 m length, 0.25mm ID, and 0.25 μm film thickness (Phenomenex, U.S.A.). The temperature program started at 60 °C followed by temperature gradient of 17 °C .min^−1^ to 155 °C and second ramp with 2.5 °C.min^−1^ to 255 °C. Finally, the baking temperature between individual analyzed samples was reached by the heating the column to 275 °C. Temperature of the transfer line was set to 250 °C, ion source temperature was held at 200 °C. The flow rate of the carrier gas (helium) was 1.2 mL·min^−1^. The full scan spectra in the range of relative mass m/z 50–450 Da were scanned. The qualitative analyses of fatty acids methyl esters were performed using external Bacterial Acid Methyl Ester (BAME) Mix (Supelco, Sigma-Aldrich, Czech Republic).

### 2.4. Sample preparation

For *in vivo* microspectroscopic experiments with spatially immobilized algal cells, 2–4% w/v solution of low temperature melting agarose (Sigma, TypeX1) in deionized water was mixed with 10–30% v/v of algal suspension directly on a microscope coverslip and covered with another coverslip. Care was taken to introduce the algae to the agarose gel 2–3 minutes before it solidified, in order to avoid unnecessary heat stress on the cells. Starting approximately 10 minutes after the immobilization, the cells were analyzed with Raman microspectroscopy.

### 2.5. Raman microspectroscopy

**Figure 2.**
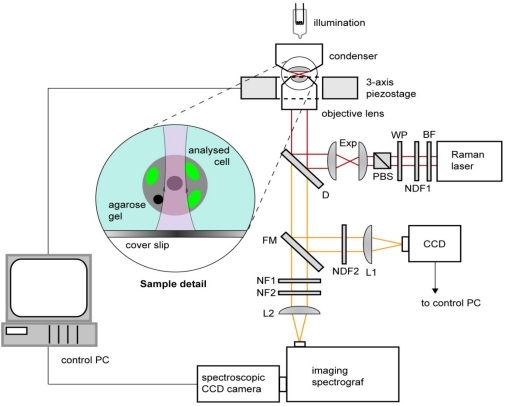
Schematic diagram of the experimental setup for Raman microspectroscopy. BF––bandpass filter, D––dichroic mirror, Exp––beam expander, FM––flipping mirror, L1,2––lenses, NDF1,2––neutral density filters, NF1,2––notch filters, PBS––polarizing beam splitter, WP––lambda-half wave plate. Inset shows the detail of the studied sample.

Raman microspectroscopic experiments with living algal cells were carried out using a home-built experimental system based on a custom-made inverted microscope frame. The layout of this system is shown in Figure 2. In order to minimize the background fluorescence emission from the studied cells as well as to minimize potential photodamage of the cells, we chose the operation wavelength of the Raman excitation laser in the near-infrared spectral region. The Raman laser beam (Ti:Sapphire, λ = 785 nm, beam diameter 0.6 mm; 899-01, Coherent) was delivered to the setup by an optical fiber that also expanded the beam diameter by a factor of 3 (not shown in the picture). Immediately after exiting from the fiber, the beam passed through bandpass filter BF (transmission bandwidth 3 nm centered on 785 nm; MaxLine LL01-785, Semrock) in order to clean up the excitation laser line. Beam diameter was further enlarged by 2× beam expander Exp before coupling to the objective lens via dichroic mirror D (LPD01-785RS, Semrock). The power of the Raman laser beam could be gradually adjusted by neutral density filter NDF1 with continuously variable optical density (Thorlabs) and by a combination of a λ/2 waveplate WP with a polarizing beam splitter cube PBS. Maximal laser power available for excitation was estimated to be approximately 60 mW at the specimen.

The Raman excitation beam was focused on the specimen with an IR-optimized water-immersion objective lens (Olympus UPLSAPO 60×, NA 1.20). This lens has a very good transmission in the near-IR spectral region and a long working distance that allowed us to work up to 200 μm deep into the specimen without a significant influence on the quality of the spectroscopic measurements. The lens was mounted on a custom-made aluminum frame that also provided a stable support for condenser and illumination light source and for 3-axis piezo-driven stage (P-517.3CL, Physik Instrumente) which served for nanometer-precise positioning of the sample relative to the objective lens. In our experiments, the cells were immobilized in agarose gel placed between standard microscope coverslips (see above). This mounting procedure allowed us to select a target cell within the specimen, focus the Raman beam on a well-defined intracellular location, and keep the cell stationary during the spectrum acquisition. For recording the calibration spectra with pure fatty acids, a small droplet of the substance was placed directly on top of a cover slip and the Raman excitation beam was focused inside the droplet approximately 20 μm away from the liquid-glass interface.

The full axial extent (depth) Δz of the excitation region was previously measured to be approximately 4 μm [27]. This value is comparable with the diffraction limit Δz_theor_ expected for focusing λ = 785 nm light with an NA = 1.2 microscope objective in water (refractive index n = 1.33) in the wide-field configuration: Δz_theor_ = 4λn/NA^2^ ≈ 3 μm [28]. Thus, we can expect the full lateral extent (width) Δx of the excitation region also attains the diffraction-limited value Δx = 1.22λ/NA ≈ 0.8 μm.

Raman scattering spectra from the target cellular compartment were collected by the objective lens and subsequently focused by lens L2 into the entrance slit of an imaging spectrograph (focal length 300 mm, f/3.9; SpectraPro 2300i, PI Acton). Two edge filters EF1 (ZX000626, Iridian) and EF2 (LP02-785RS, Semrock) were placed in the detection light path to prevent the excitation light from reaching the spectrograph. The Raman scattered light was dispersed with a 600 gr/mm diffraction grating, imaged on the chip of a high-sensitivity liquid-nitrogen-cooled spectroscopic CCD camera (Spec-10:100BR/LN, Princeton Instruments), and recorded using the camera control software (WinSpec). Recorded spectra were processed off-line using custom-written routines implemented in Matlab software (MathWorks). In order to facilitate the observation of the specimen and select the target location within the studied cell, light in the imaging path could be diverted via flipping mirror FM to a standard CCD camera connected to a monitor and a PC controlling the experiment.

### 2.6. Spectrum processing and analysis

Despite using a near-infrared laser beam for the Raman scattering excitation and focusing the Raman probe specifically into the lipid storage bodies, the collected Raman spectra typically still display a noticeable non-specific fluorescence background. A major part of this background comes from chlorophyll autofluorescence that is emitted at wavelengths much longer than the dominant emission bands of the pigment (685 nm, 695 nm, and *ca.* 730 nm) due to the excitation with a 785 nm beam. [29]. The fluorescence emission contributes by a spectral signal monotonously falling with increasing wavenumber and varying from location to location in a single cell. Besides the pigment autofluorescence, glass coverslip contributes a broad peak (width ∼500 cm^−1^) centered at ∼1,400 cm^−1^ whose intensity depends on the distance from the coverslip surface. The overall nonspecific background of varying intensity and shape hampers the quantitative analysis of the heights of selected Raman spectral peaks.

In order to extract quantitative information from the experimentally obtained spectral data, we adopted the Rolling Circle Filter (RCF) technique for background removal [30]. In principle, RCF is a high-pass signal filter that allows separating the narrow Raman spectral peaks from the background whose radius of curvature is significantly higher. With an appropriate choice of the filter parameters (filter width and number of filter passes), background can be effectively removed without causing a significant distortion of the signal peaks. When multiple spectra from different specimens are analyzed and compared, it is essential to keep the filter parameters constant for the whole spectral series and, thus, keep the residual distortion of the spectral peaks at a similar level. All the spectra presented in this paper have been corrected with the above described procedure.

**Figure 3.**
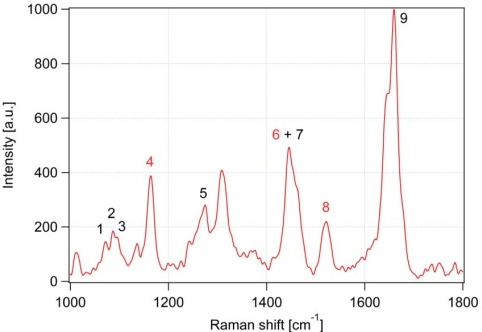
Typical Raman scattering spectrum of *Trachydiscus minutus*. Individual numbered bands are assigned in Table 1. Raman bands 7 and 9 are used to calculate the degree of lipid unsaturation. Spectrum acquisition parameters: integration time 20 s, laser power at the specimen 13 mW. Red numbers indicate β-carotene vibrations.

After the background removal, the actual analysis of the Raman spectra can be carried out. Figure 3 shows a typical background-corrected Raman spectrum obtained from a lipid body inside a *Trachydiscus minutus* cell. The most prominent Raman spectral features observed in the spectrum are summarized in Table 1.

In our experiments, we determine the ratio of unsaturated-to-saturated carbon-carbon bonds in algal lipid molecules. To this end, we employ two specific spectral peaks ν_U_ at 1,656 cm^−1^ (*cis* C═C stretching mode proportional to the amount of unsaturated C═C bonds, peak No.9 in Table 1 and Figure 3) and ν_S_ at 1,445 cm^−1^ (CH_2_ scissoring mode proportional to the amount of saturated C-C bonds, peak No.7 in Table 1 and Figure 3). We found these peaks free of any significant interference or overlaps with Raman signals of other cellular components (see the discussion below). Both of these peaks are well documented as being very strong in Raman spectra of lipids [19]. From the ratio ν_U_/ν_S_, the average ratio of double-to-single carbon-carbon bonds N_C═C_/N_CH2_ in the specimen – specimen mass unsaturation - can be estimated. This can be accomplished by recording a calibration curve that relates ν_U_/ν_S_ to N_C═C_/N_CH2_ for pure fatty acids of varied degree of the hydrocarbon chain unsaturation. On the basis of the published iodine values for the fatty acids used in our calibration [31], it is possible to directly convert the measured values of N_C═C_/N_CH2_ to the iodine values for a given sample.

## 3. Results and Discussion

### 3.1. Calibration of iodine value against spectroscopic data

**Figure 4.**
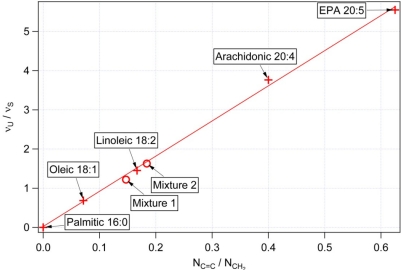
Dependence of the observed ratio ν_U_/ν_S_ of the Raman spectral peaks at 1656 cm^−1^ and 1445 cm^−1^ on the molecule mass unsaturation N_C═C_/N_CH2_ Crosses mark experimental data obtained with pure fatty acids, straight line is a fit of this data. Circles indicate verification data points obtained with mixtures of oleic and arachidonic acids of different molar ratios; this data was not included in the fit.

**Figure 5.**
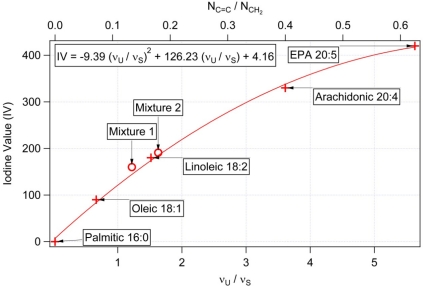
Calibration curve for estimating the iodine value (IV) from the Raman spectral data. The IV range of 0–430 is covered which includes virtually all biologically relevant fatty acids. Crosses mark experimental data obtained with pure fatty acids, continuous line is a parabolic fit of this data. Circles indicate verification data points obtained with mixtures of oleic and arachidonic acids of different molar ratios; this data was not included in the fit. Formula given in the top left corner of the graph was used for calculating IV of the studied algae from experimental spectroscopic data.

In order to link the experimentally observed values of ν_U_/ν_S_ to the values of mass unsaturation ratio N_C═C_/N_CH2_ and the actual iodine values (IV), we performed a series of Raman spectroscopic measurements of pure fatty acids of varied degree of unsaturation (see Table 2 for the summary of the samples used). The results of these measurements are shown in figures 4 and 5.

As expected, the obtained values of ν_U_/ν_S_ are directly proportional to the calculated values of N_C═C_/N_CH2_ (Figure 4) for individual fatty acids because the heights of the spectral peaks ν_U_ (ν_S_) scale linearly with the numbers N_C═C_ (N_CH2_) of C═C (CH_2_) groups per molecule. In contrast, the dependence of IV on both ν_U_/ν_S_ and N_C═C_/N_CH2_ deviates from linearity for IV > ∼150 (Figure 5). This stems from the fact that IV is proportional to the number of C═C bonds per the total length of the molecule including both C═C and CH_2_ groups. For fatty acids with small degree of unsaturation we can assume that N_CH2_ ≫ N_C═C_ and, consequently, N_C═C_/N_CH2_ ≈ N_C═C_/(N_CH2_ + N_C═C_). As the number of C═C groups per molecule grows, the two ratios start to diverge thus causing the observed non-linearity. The calibration curve presented in Figure 5 can be employed to relate the Raman spectroscopic data the corresponding iodine values of the studied sample.

To verify the validity of the calibration curve also for mixtures of different fatty acids, we performed additional spectroscopic measurements with binary mixtures of oleic and arachidonic acids of two different molar ratios: “Mixture 1” (6.1 mg of oleic acid, 4.8 mg of arachidonic acid), and “Mixture 2” (10.7 mg of oleic acid, 4.8 mg of arachidonic acid). Mass unsaturation and IV of the mixtures were calculated from the known molar ratios and the respective values of IV and N_C═C_/N_CH2_ of the two components. The obtained pairs of values [N_C═C_/N_CH2_, ν_U_/ν_S_] and [ν_U_/ν_S_, IV] for both analyzed mixtures fall on the calibration curves of Figures 4 and 5. Thus, we conclude that these calibration curves can be adopted for characterizing average mass unsaturation and the iodine value in the studied algal cells.

### 3.2. Determination of iodine value of lipids in living algal cells

We investigated the fatty acid composition of lipid storage bodies in three algal species––*Trachydiscus minutus, Botryococcus sudeticus*, and *Chlamydomonas sp.* Before the spectroscopic experiments, the living cells were immobilized in agarose (see Materials and methods). Subsequently, Raman scattering spectra were recorded over the spectral range of 300–2,000 cm^−1^. For the analysis of the algal fatty acid composition, we focused on the spectral range from 1,000 cm^−1^ to 1,800 cm^−1^ that contained the most relevant information.

In order to obtain Raman scattering spectra with a good signal-to-noise ratio, we used acquisition times in the range of 10–20 s and excitation power approximately equal to 15 mW at the specimen plane. Assuming the laser beam was focused to a diffraction limited spot of ∼0.8 μm in diameter, the corresponding photon flux density was 2 × 10^11^ μmol(photons)·m^−2^·s^−1^. This value is by orders of magnitude higher than the physiological irradiance level. Even though we used infrared excitation photons, which are not efficiently absorbed by the cellular photosystems, we can expect the laser beam irradiation influences the photosystem states and possibly causes their irreversible damage. Indeed, when the Raman probe beam is focused into a chloroplast inside a cell, the recorded spectral time series show a monotonous decrease of the background fluorescence caused by the cellular pigment bleaching (data not shown). A similar trend was observed by Huang *et al.* who used a green excitation beam for recording the Raman scattering spectra of algae [16]. However, when the Raman beam is focused selectively into a lipid body, the rate of change of the observed fluorescence background is significantly slower. Thus, the selective targeting of intracellular compartments with spatially resolved Raman microspectroscopy can minimize the impact of the spectroscopic measurement on the physiological state of cells under investigation.

**Figure 6.**
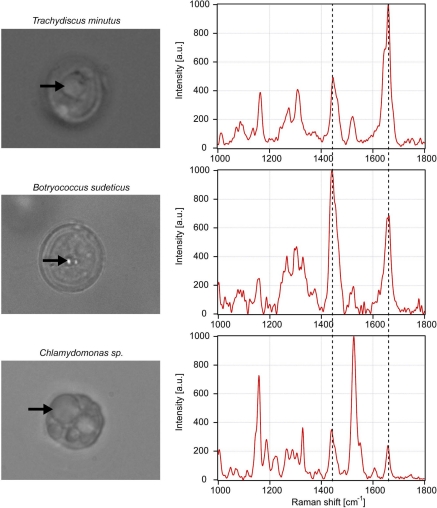
Typical Raman scattering spectra of intracellular lipid bodies contained in three different algal species: *Trachydiscus minutus* (top), *Botryoccocus sudeticus* (middle), and *Chlamydomonas sp* (bottom). Raman bands used for the calculations of iodine value are highlighted with dashed vertical lines. Corresponding pictures to the left of the spectra show the lipid bodies from which the spectra were recorded (indicated by the black arrows).

Figure 6 shows typical Raman scattering spectra obtained from intracellular lipid bodies in the three studied algal species. It is clearly visible that the ratios ν_U_/ν_S_ of the Raman spectral peaks at 1,656 cm^−1^ and 1,445 cm^−1^ differ for individual species; specifically *Trachydiscus minutus* has a significantly higher content of the unsaturated fatty acids in comparison with the other two species. Table 3 summarizes the results of the repeated spectroscopic measurements carried out with algae together with the estimates of their iodine values obtained from the calibration curve of Figure 5. The observed cell-to-cell variability within each studied species indicates differences in the composition and concentration of fatty substances. This variability can be attributed mainly to the different growth phases of the individual cells included in the analysis and to the small variations of cultivation conditions (concentration of nitrate, phosphate, and other ions, intensity of lighting, and cultivation temperature) [32–34]. The average iodine values and their standard deviations for the three algal species included in our study are (216 ± 7) for *Trachydiscus minutus*, (88 ± 5) for *Botryococcus sudeticus*, and (93 ± 2) for *Chlamydomonas sp*. (see Table 3).

We would like to point out that the reproducibility of the measurements for individual algal cells belonging to the same cell population was found to be sufficiently good to conclude that the measurement and evaluation procedure exploited here lend themselves to a reliable diagnostics; the Raman spectral signatures obtained from the regions inside individual cells where the lipid bodies reside characterize well the given cell in terms of the lipid content and composition. Currently, we are investigating in more detail the influence of the location/size of the excitation region on the obtained spectra and the contribution of the other parts of the cell (e.g., plastids, organelles, and phospholipid membranes) to the observed spectral features.

In a real-world application, it is possible (and, in fact, quite likely) that the cell population contained within the studied culture (bioreactor or cultivation pond) can consist of several metabolically distinct sub-populations or even contaminants. In such a case, the presented microspectroscopic approach can identify different individuals within the batch and, thus, it can serve as a base for the cell selection and sorting. Systematic studies are still required to establish a spectroscopic method for a rapid and robust investigation and analysis of large, potentially inhomogeneous samples and to determine the limitations associated when evaluating an entire culture. Such studies exploiting the limitations of the Raman spectroscopy technique are currently under way in our laboratories. Our first results presented here pave the way for more extensive investigations involving the effect of the growth conditions on the observed spectral signatures and spectroscopic analysis of additional algal species.

We verified the data obtained from Raman microspectroscopic measurements on *Trachydiscus minutus* by comparing them with GC-MS analysis of the algal fatty acid composition. As mentioned above, GC-MS is a well established technique that provides a robust benchmark for our results. To calculate the mean IV of the fatty acid mixture from the GC-MS results, we determined the average mass unsaturation ratio N_C═C_/N_CH2_ from the data presented in Table 4 and, subsequently, employed the calibration curve of Figure 5. We found an excellent agreement between the two methods of IV determination: analyses based on Raman spectroscopy gave IV = 216 for *Trachydiscus minutus* while the GC-MS based calculations gave IV = 194. The difference between obtained IV (Raman spectroscopy) and GC-MS results can be explained by the slightly different growth conditions because these cultures were cultivated separately in the two different laboratories. We carried out a similar comparison for *Botryococcus sudeticus*, where we compared our spectroscopic data with the fatty acid composition of *Botryococcus braunii* published by Tran *et al.* [18]. We found again a reasonable agreement between both methods (IV = 88 from the Raman spectra and IV = 118 from GC-MS). Here, the difference can be attributed to the to the different strain of Botryococcus used by Tran *et al.* [18] and the different growth condition. Hence, we conclude that the *in vivo* obtained Raman spectroscopic data can indeed serve as a robust and reliable indicator of the iodine value of algal storage lipids.

### 3.3. Influence of carotene on the iodine value determination

**Figure 7.**
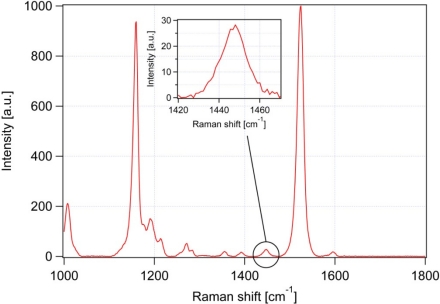
Raman scattering spectrum of β-carotene. The inset shows the detail of β-carotene band at 1,442 cm^−1^.

It is evident from the spectra presented in Figure 6 that lipids are not the only molecules contributing to the overall Raman spectrum acquired from a lipid body. Among various possible intracellular compounds, β-carotene plays the most important role as its NIR-excited Raman spectrum can be significantly enhanced due to an electron-phonon coupling mechanism [14]. Moreover, β-carotene is soluble in lipids and, thus, its concentration in the lipid bodies is expected to be higher than the cellular average. β-carotene has two very strong spectral peaks at 1,157 cm^−1^ and 1,525 cm^−1^ (see Figure 7 and Table 1) that do not interfere with the lipid bands in the composition analysis. Besides these two strong peaks, however, there is also a much weaker β-carotene peak present at 1,442 cm^−1^ that overlaps with the CH_2_ scissoring mode of lipids at 1,445 cm^−1^ (Table 1). This peak can potentially cause a bias in the iodine value estimations.

To quantify the possible influence of β-carotene on IV measurement, we recorded the Raman scattering spectrum of pure β-carotene which is shown in Figure 7. From this spectrum, the ratio of the β-carotene peaks at 1,442 cm^−1^ and 1,525 cm^−1^ can be determined. We selected the peak at 1,525 cm^−1^ as a reference because it is the dominant carotene peak that is most easily visible in the Raman scattering spectra acquired from cells. The calculated ratio of the two carotene peaks is about 1/35; hence, β-carotene influence might be non-negligible for some of the measurements on algae where the dominant β-carotene transitions are strongly visible.

Inspection of the live-cell spectra presented in Figure 6 shows that this is indeed the case for the IV estimation of *Chlamydomonas* species. Consequently, we included the necessary correction to the IV estimation: contribution of β-carotene at 1,442 cm^−1^ calculated from the experimentally measured peak height of the β-carotene peak at 1,525 cm^−1^ multiplied by the peak ratio 1/35 was subtracted from the measured peak height at 1,445 cm^−1^ for CH_2_ group. This correction has already been included in Table 3 and resulted in an average increase of the ratio ν_U_/ν_S_ of about 0.06 corresponding to an increase of IV of about 8. Apart from *Chlamydomonas* corrections were not necessary for the other studied species because the contribution of the β-carotene vibration was negligible (see Figure 6).

## 4. Conclusions

In this paper we have demonstrated the potential of Raman microspectroscopy for the fast and spatially resolved characterization of the composition of selected intracellular regions in individual living algal cells. In particular, we have focused on lipid storage bodies and quantified the degree of unsaturation of algal lipids (iodine value) which is an important parameter for bio-fuel production and food industry. To this end, we have employed the intensity of characteristic Raman spectral peaks corresponding to the saturated and unsaturated carbon-carbon bonds in lipid molecules. On the basis of the calibration data obtained with pure fatty acids of varied degree of unsaturation, we have calculated the average ratio of unsaturated-to-saturated carbon-carbon bonds in algal lipids and determined their average iodine value. We have shown that various algal species display significantly different iodine values (88 up to 216), which has important implications for biotechnological exploitation of the particular algae. Specifically, our results suggest that *Trachydiscus minutus* with its very large iodine value (∼216) might not be the species of choice for the biofuel production where iodine values below 120 are typically exploited. However, this alga might excel in the food industry applications where high amounts of unsaturated fatty acids, rich in Omega-3, are required. Our spectroscopic data agree well with the results obtained by gas chromatography that can be considered as an experimental standard for characterizing the fatty acid composition of cells.

Variation of the iodine values determined for individual cells belonging to the same cell population is on the order of a few per cent. However, this variation does not represent the fundamental resolution limit of our technique as it stems mostly from the differences in the physiological state of the studied cells. In principle, the sensitivity of the Raman microspectroscopic measurements can be employed for identifying different sub-populations within the analyzed algal population. Acquisition of the Raman spectra can be optimized in order to minimize the impact on the physiological state of the studied cells. Thus, we have found Raman microspectroscopy to be a fast, versatile, and virtually non-invasive tool for applications in algal lipid engineering and industry.

## Figures and Tables

**Table 1.** Summary of the most prominent peaks / bands observed in the Raman spectra of algae, together with suggested assignments of vibration modes and contributing chemical compounds. Peak numbers of the table are used to identify features in the spectra shown in Figure 3.

Peak #	Raman feature (in cm^−1^)	Suggested assignment

1	1,060	C-C skeletal stretching vibration, out-of-plane
2	1,085	C-C skeletal stretching vibration; *gauche* chain conformer
3	1,125	C-C skeletal stretching vibration; *trans* chain conformer, in-plane
4	1,157	β-carotene
5	1,267	*cis* double bond ═C-H bend’ in plane
6	1,442	β-carotene
7	1,445	CH_2_ bend, scissoring deformation; **saturated fat indicator**
8	1,525	β-carotene
9	1,656	*cis* C═C stretching vibration; **unsaturated fat indicator**

**Table 2.** Fatty acids used for the calibration of Raman spectral data against the actual mass unsaturation of the molecules and their iodine values. Raman scattering spectra of fatty acids were acquired with 10 s integration time and excitation power of ∼13 mW at the specimen.

	**N_C═C_ Number of double bonds per molecule**	**N_CH2_ Total number of CH_2_ groups per molecule**	**N_C═C_/N_CH2_ Mass unsaturation**	**IV Iodine values [31]**	**υ_U_/υ_S_ Raman intensity at 1656 cm^−1^/1445 cm^−1^**
Palmitic **16:0**	0	14	0	0	<0.03
Oleic **18:1**	1	14	0.071	90	0.65
Linoleic **18:2**	2	12	0.166	180	1.46
Arachidonic **20:4**	4	10	0.4	330	4.18
EPA (Eicosapentaenoic acid) **20:5**	5	8	0.625	420	5.61

**Table 3.** Measured ratios ν_U_/ν_S_ of the Raman spectral peaks at 1,656 cm^−1^ and 1,445 cm^−1^ for the three studied algal species. Corresponding iodine values are calculated from the formula presented in Figure 5. SD––standard deviation of the data obtained from four different algal cells analyzed for each algal species.

	*Trachydiscus minutus*	*Botryococcus sudeticus*	*Chlamydomonas sp.*
Algal cell 1	1.91	0.74	0.76
Algal cell 2	1.9	0.73	0.73
Algal cell 3	2.01	0.67	0.74
Algal cell 4	2.07	0.66	0.76
Averaged value ν_U_ /ν_S_	1.97	0.70	0.75
SD ν_U_ /ν_S_	0.081	0.040	0.015
Estimated IV (equation from Figure 5)	216	88	93
SD IV	7	5	2

**Table 4.** Fatty acid composition of the lipid fraction of *Trachydiscus minutus* determined from a GC-MS analysis. Molar ratios *n*_i_ of the individual fatty acids in the algal lipid fraction are used to determine the average mass unsaturation ratio N_C═C_/N_CH2_ of the mixture as Σ(*n*_i_ N_C═C_^i^)/Σ(*n*_i_ N_CH2_^i^). For the data given below, the actual value of N_C═C_/N_CH2_ = 0.181 which corresponds to IV = 194.

**Fatty acids**	**Content (% w/w)**	**Molecular weight**	**Molar ratio***n*_i_	**N_C═C_**^i^	**N_CH2_**^i^

Myristic	5.13	228.37	0.065	0	12
Palmitoleic	7.48	254.41	0.085	1	12
Palmitic	9.9	256.42	0.111	0	14
Linoleic	12.95	280.45	0.133	2	12
Oleic	9.12	282.46	0.093	1	14
Stearic	1.99	284.48	0.020	0	16
Arachidonic	8.54	304.5	0.081	4	10
EPA	30.25	302.45	0.289	5	8
Behenic	11.58	340.58	0.098	0	20
Lignoceric	3.06	368.63	0.024	0	22
